# Case report: Treatment with ensartinib shows good response to SQSTM1-ALK fusion in lung adenocarcinoma

**DOI:** 10.3389/fphar.2024.1433894

**Published:** 2024-11-01

**Authors:** Pandeng Wang, Zhuo Jiang, Jianji Guo, Tao Liu, Zhen Liu, Dongdong Wang, Honglin Li

**Affiliations:** ^1^ Department of Cardiothoracic Surgery, The First Affifiliated Hospital of Guangxi Medical University, Nanning, Guangxi, China; ^2^ Shanghai Yinji Technology Co., Ltd., Shanghai, China; ^3^ Shanghai Zhongyou Inspection & Medical Co., Ltd., Shanghai, China

**Keywords:** SQSTM1-ALK, rearrangement, lung cancer, targeted therapy, ensartinib

## Abstract

Lung cancer is a prevalent malignancy, with the rearrangement of the anaplastic lymphoma kinase (ALK) gene being responsible for a minority of cases of non-small cell lung cancer (NSCLC). NSCLC patients harboring ALK fusion proteins demonstrate sensitivity to ALK tyrosine kinase inhibitors (TKIs). In this report, we describe the case of a female patient with metastatic lung adenocarcinoma, identified through NGS to carry a rare inverted SQSTM1-ALK (S5, A20) fusion. The patient received ensartinib as first-line therapy, resulting in a partial response (PR). At the time of publication, the patient’s condition remained favorable. We have, for the first time, identified the presence of SQSTM1-ALK fusion in pericardial effusion, with the favorable response to ensartinib validating the oncogenic potential of SQSTM1-ALK fusion. The substantial advancements and extensive utilization of NGS have facilitated the identification of rare fusion variants.

## Introduction

Lung cancer is a prevalent malignancy, and those with specific molecular genetic alterations in the ALK, BRAF, or EGFR genes have been endorsed by the US Food and Drug Administration (FDA) as first-line targeted therapies. Rearrangement of the anaplastic lymphoma kinase (ALK) gene is observed in 2%–7% of NSCLC patients, and is particularly frequent in younger individuals, non-smokers, and those with adenocarcinoma (Kwak et al., 2010). NSCLC patients harboring ALK fusion proteins demonstrate sensitivity to ALK tyrosine kinase inhibitors (TKIs).

Approximately half of the cases are diagnosed at an advanced, metastatic stage. Advanced lung cancer may result in the occurrence of pericardial effusion. Due to the close relationship between the cardiovascular and pulmonary systems, it is common for patients with advanced lung cancer to exhibit pericardial effusion, bloody pericardial effusion, and metastasis of cancer cells. Typically, only a restricted amount of biopsy material is available for companion diagnosis using NGS panels. In this report, we depict a case study of a patient with metastatic lung adenocarcinoma who declined direct tumor biopsy sampling and instead opted for pericardial effusion extraction for diagnostic purposes. Subsequent testing revealed the presence of SQSTM1-ALK fusion. Subsequent to treatment with ensartinib, the ailment was accurately diagnosed via initial limited samplings, thereby obviating the need for multiple rebiopsies.

## Case description

**FIGURE 1:**
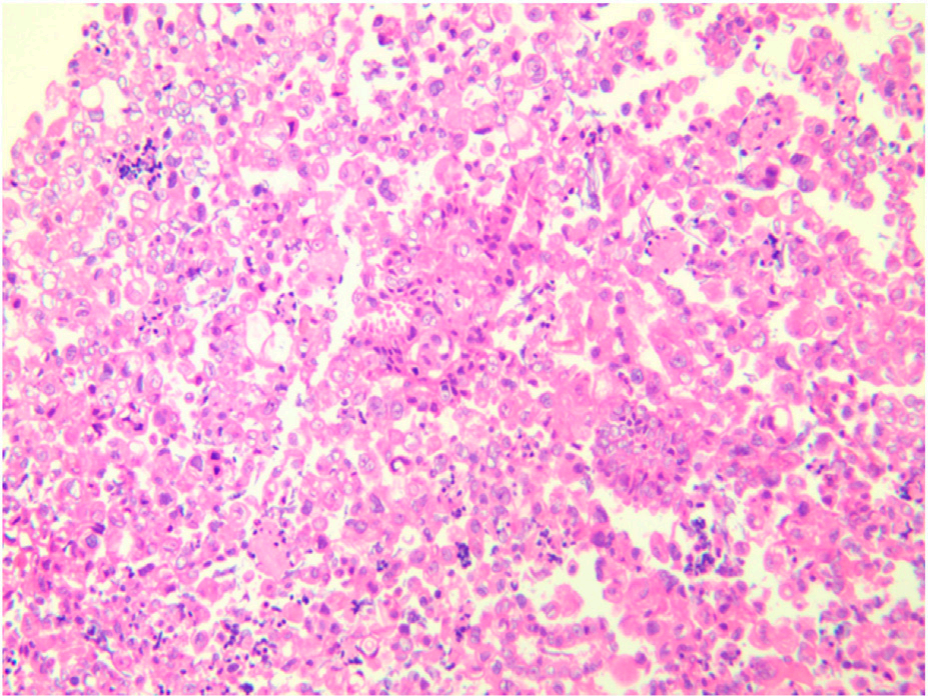
Tumor cell content.

**FIGURE 2:**
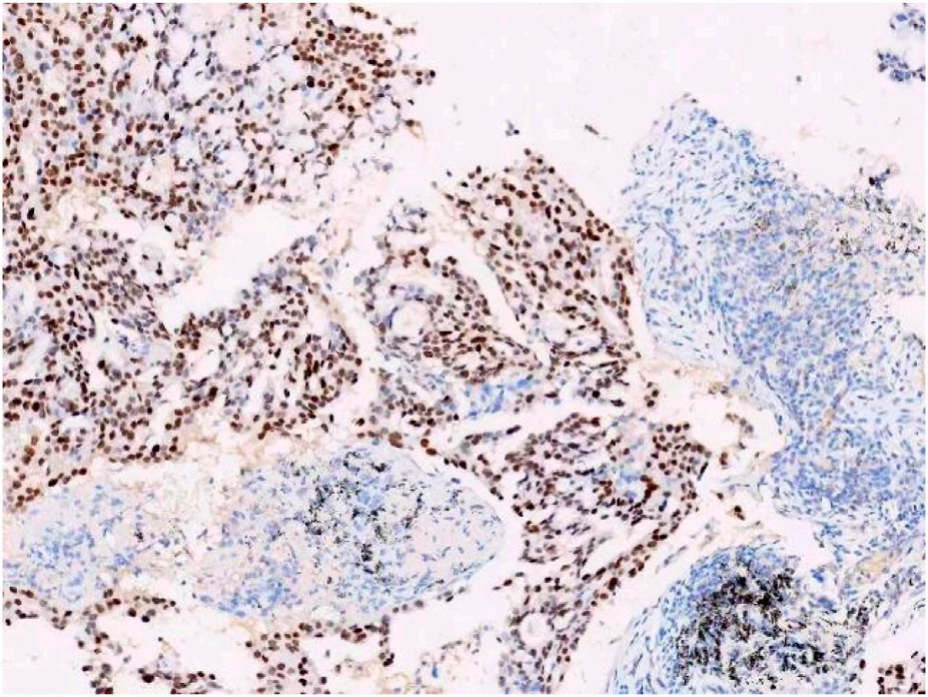
TTF1 immunohistochemical results.

**FIGURE 3:**
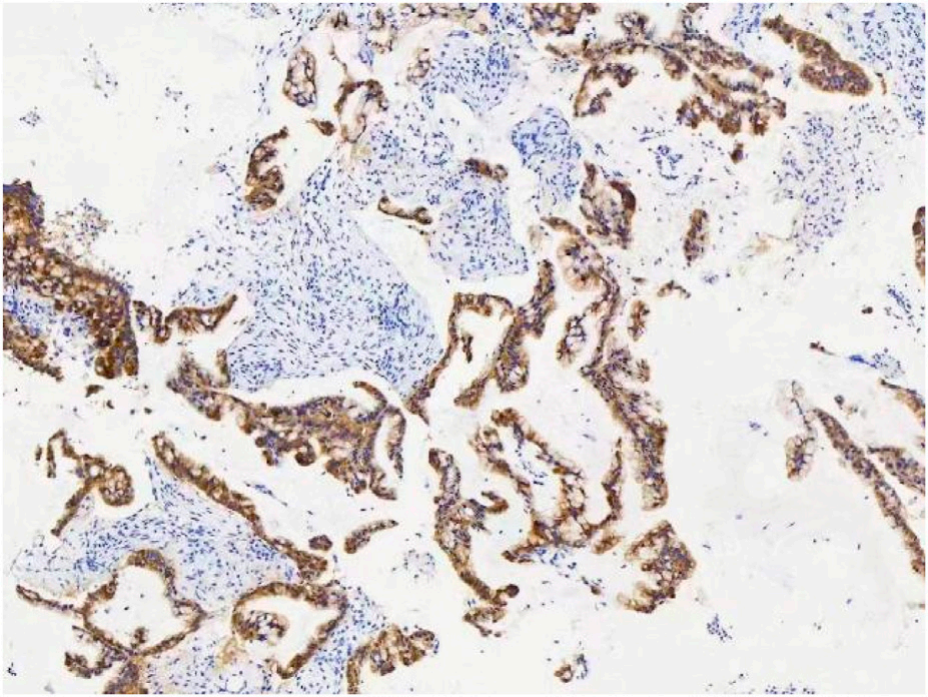
ALK immunohistochemical results.

A female patient in her 60s, who is Chinese and of Zhuang nationality, with a junior high school education level, married status, a farmer by occupation and in poor economic condition, presented with chest pain accompanied by dyspnea and was diagnosed with advanced metastatic lung adenocarcinoma upon examination. The patient had no history of smoking and alcohol consumption. Neither her parents nor her siblings have a history of lung cancer. The clinical stage was T2bN2M1a, with no extrathoracic metastases detected upon radiological follow-up. Due to the patient’s decision to decline a lung tumor biopsy, pericardial effusion was obtained for HE staining, which revealed that tumor cells comprised 85% of the sample (Figure 1). TTF1 immunohistochemistry results confirmed that the malignant pericardial effusion originated from metastasis of lung adenocarcinoma (Figure 2). The ALK immunohistochemistry test also showed positive results (Figure 3). Following the confirmation of pericardial effusion as tumor metastasis, DNA of tumor cells was extracted from the effusion for genetic testing.

**FIGURE 4:**
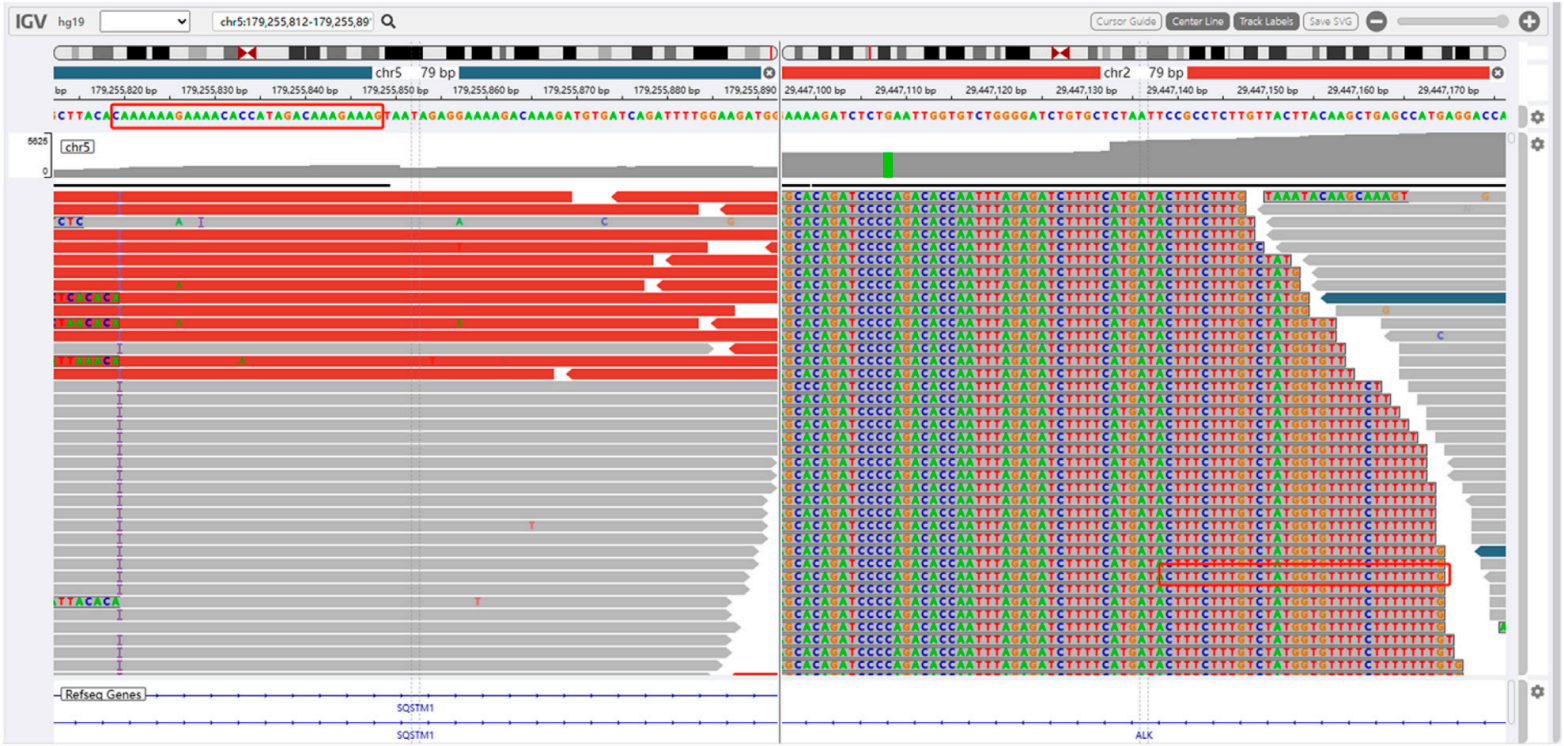
Breakpoint of SQSTM1 gene.

**FIGURE 5:**
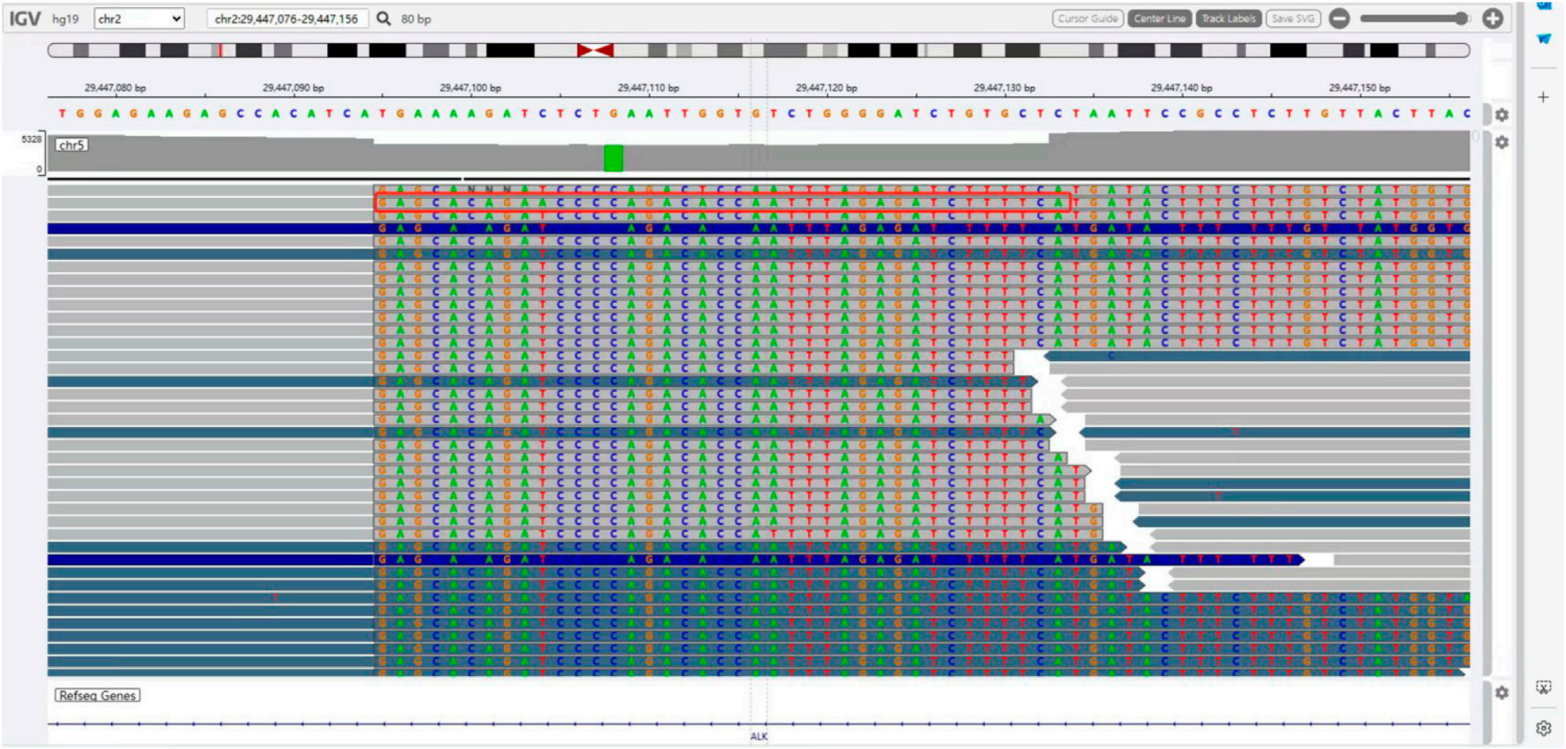
Inversion of 39BP base at breakpoint of ALK gene.

**FIGURE 6:**
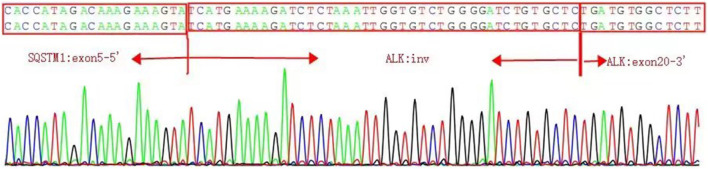
SQSTM1-ALK fusion.

The presence of SQSTM1-ALK in the pericardial effusion sample was validated through next-generation DNA sequencing (NGS) using a 160-gene panel provided by Shanghai Zhongyou Inspection & Medical Co., Ltd. The genetic testing report (using the GenarsR system from Shanghai Yinji Technology Co., Ltd) revealed the location of the SQSTM1 breakpoint at Exon5 chr5:179255851, and the ALK breakpoint at Exon20 chr2:29447094. A 39-base pair inversion of the ALK gene was observed at the breakpoint location, as illustrated in Figures 4, 5. The patient was also found to harbor an unidentified MSH6 S346C mutation. This fusion has not previously been documented in cases of lung adenocarcinoma, and the Sanger verification results are provided in Figure 6.

**FIGURE 7:**
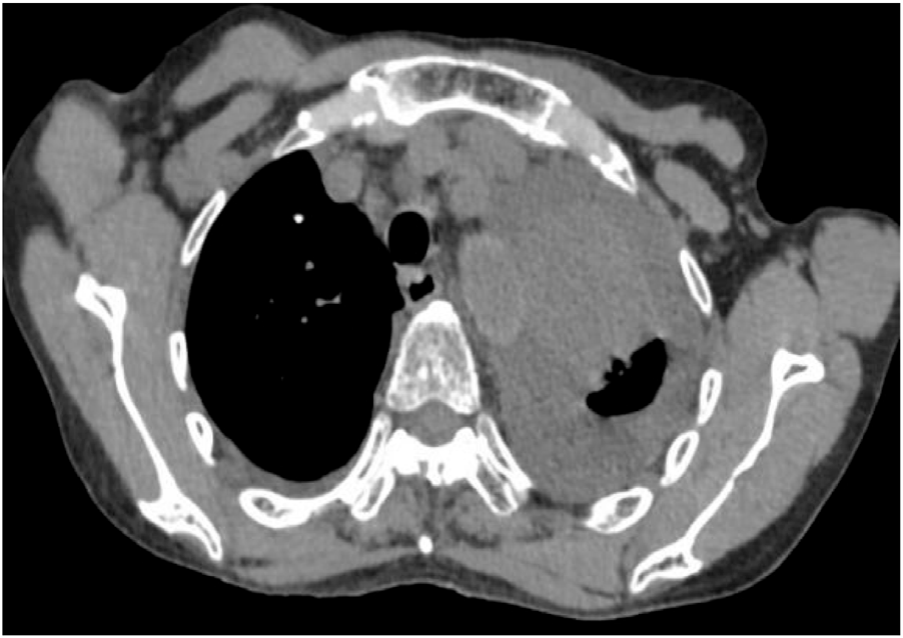
Tumor condition before treatment.

**FIGURE 8:**
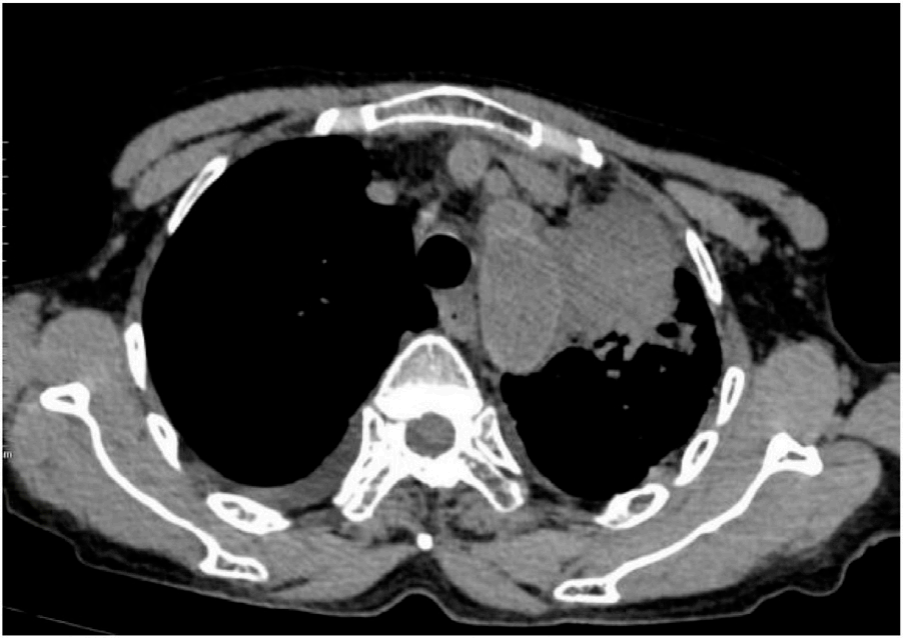
Tumor size after 1 month of treatment.

**FIGURE 9:**
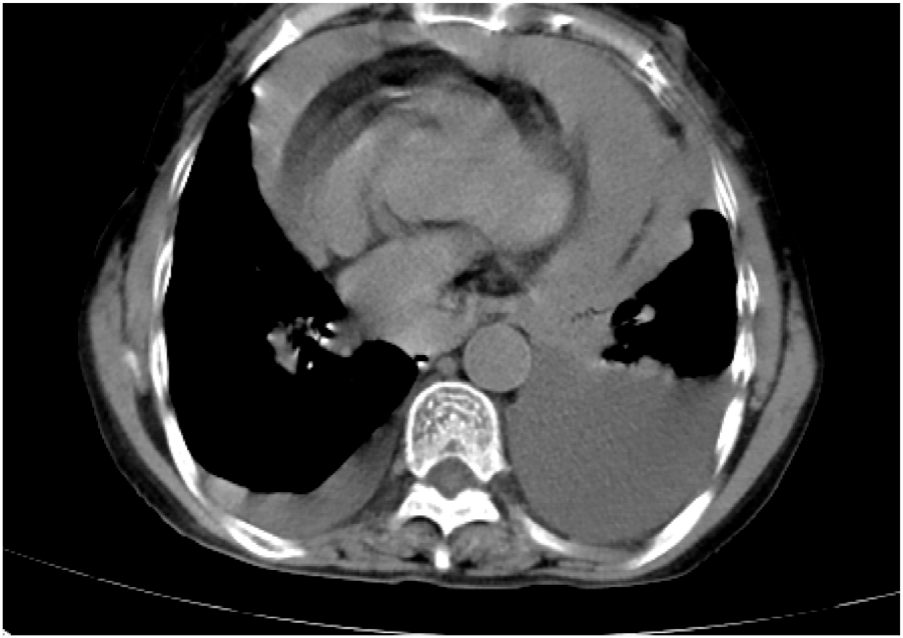
Pericardial effusion condition before treatment.

**FIGURE 10:**
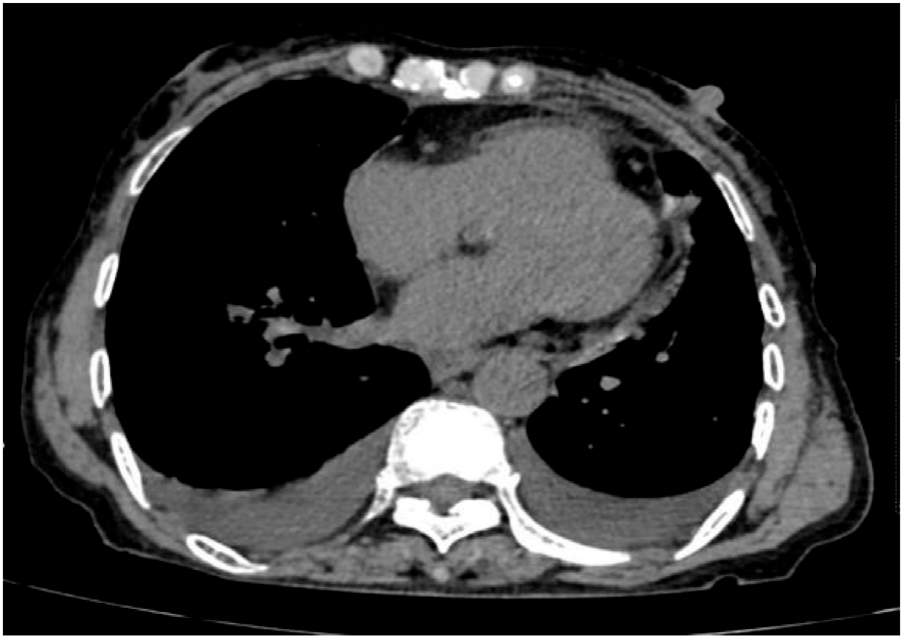
Pericardial effusion and pleural effusion after 1 month of treatment.

**FIGURE 11:**
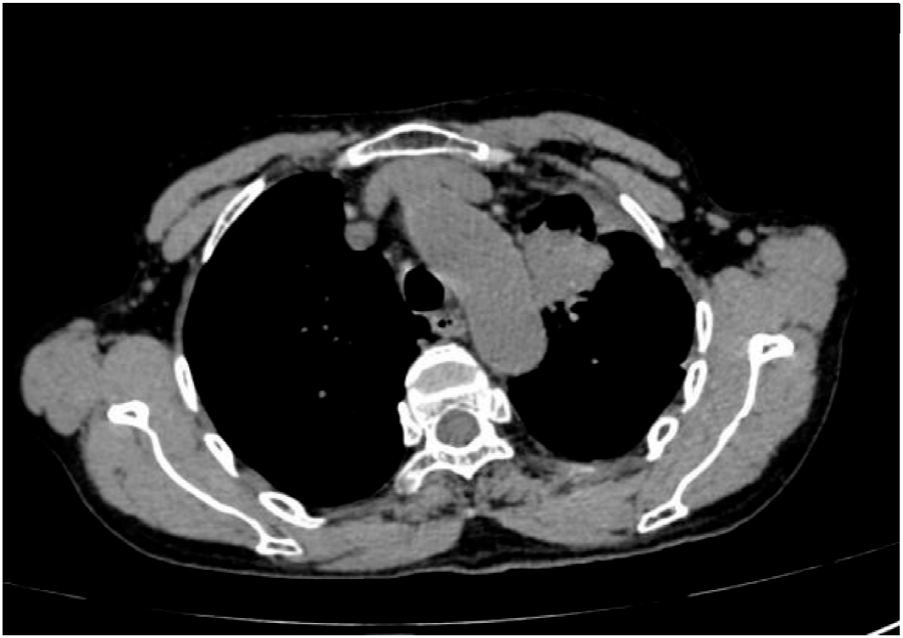
Tumor size after 6 months of treatment.

**FIGURE 12:**
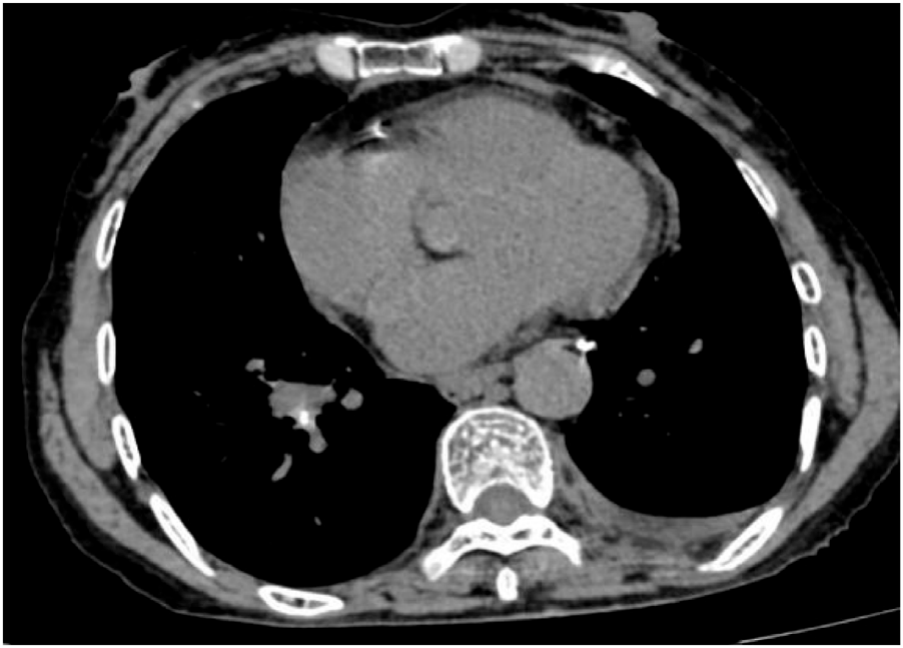
Pericardial and pleural effusion after 6 months of treatment.

Following treatment with ensartinib at 225 mg QD, the patient exhibited a decrease in CEA level from 104.97 ng/mL to 5.25 ng/mL, and a reduction in carbohydrate antigen 125 level from 312.50 μg/mL to 43.84 μg/mL after 1 month. Additionally, the chest CT scan revealed a reduction in tumor size (Figures 7, 8) as well as pericardial effusion (Figures 9, 10). After 6 months of treatment, the tumor size remained the same as after 1 month of treatment (Figure 11). After 6 months of treatment, the condition of pericardial effusion and pleural effusion (Figure 12).

## Discussion

In this report, we present the case of a 60-year-old female diagnosed with advanced lung adenocarcinoma harboring the rare SQSTM1-ALK fusion, which had metastasized to the pericardium. While several ALK inhibitors have been approved for ALK-rearranged non-small cell lung cancer (NSCLC), this is the first documented case of using Ensartinib to treat a patient with this specific fusion. Our findings show that Ensartinib demonstrated significant efficacy, After 1 month of treatment, the patient achieved partial response (PR), and the effect was sustained for at least 6 months without any observable toxicity. This suggests that Ensartinib may be a promising therapeutic option for lung adenocarcinoma patients with the SQSTM1-ALK fusion, though complete remission (CR) was not achieved during the observation period.

Ensartinib was chosen over other ALK inhibitors such as Crizotinib and Alectinib based on its demonstrated superiority in preclinical and early clinical trials. Ensartinib has shown a broader inhibitory profile, effectively targeting ALK mutations, including those resistant to first- and second-generation inhibitors. This makes it a suitable first-line treatment, especially for patients with brain metastases, as Ensartinib crosses the blood-brain barrier more efficiently (Wang et al., 2023). Additionally, the patient’s overall health status and multiple metastases influenced the decision to use Ensartinib, as a more comprehensive systemic control was needed. Given the limited data on the effectiveness of other ALK inhibitors in patients with SQSTM1-ALK fusion, Ensartinib presented a novel approach for this case.

Although the patient did not experience any drug-related toxicity, Ensartinib’s potential side effects should still be considered, especially in comparison to other ALK inhibitors such as Alectinib and Crizotinib. Common side effects of Ensartinib include skin rash, elevated liver enzymes, and fatigue (Zhou et al., 2023). The absence of any significant side effects in this patient is particularly noteworthy, given the advanced stage and the presence of multiple metastases. In contrast, Alectinib and Crizotinib are often associated with gastrointestinal disturbances and muscle pain, with Crizotinib also known to cause higher incidences of vision problems (Zhou et al., 2023). The good tolerability of Ensartinib in this case suggests that it could be an effective treatment for patients unable to endure the side effects of other ALK inhibitors.

The SQSTM1-ALK fusion has been identified in other cancers, such as inflammatory myofibroblastic tumors (IMT), epithelioid fibrous histiocytoma (Dickson et al., 2018), and large B-cell lymphoma (d’Amore et al., 2013). However, the disease course and treatment responses in these cancers differ from those in lung adenocarcinoma. For example, a 46-year-old male with SQSTM1-ALK-positive IMT responded significantly to Alectinib, with a 17-month progression-free survival (Honda et al., 2019). In our patient, the presence of cardiac and brain metastases at diagnosis suggested a more aggressive disease progression. This suggests that the presence of the SQSTM1-ALK fusion in lung adenocarcinoma may lead to rapid progression, emphasizing the need for prompt and aggressive treatment strategies.

The role of STAT3 inhibitors in treating SQSTM1-ALK fusion-positive cancers, as well as the combination of ALK and STAT3 inhibitors, warrants further investigation. In lymphoma cases involving SQSTM1-ALK fusion, STAT3 phosphorylation plays a key role in pathogenesis (d’Amore et al., 2013). Exploring whether this mechanism similarly contributes to lung adenocarcinoma could open new therapeutic avenues, particularly for combined treatment strategies with ALK and STAT3 inhibitors. Moreover, resistance to second-generation ALK inhibitors highlights the need to assess third-generation inhibitors such as Lorlatinib, which has shown efficacy in overcoming resistance mutations (Lin et al., 2024). Clinical trials investigating Lorlatinib in SQSTM1-ALK fusion-positive patients could provide valuable insights into the long-term management of resistant cases.

## Conclusion

This study presents the inaugural successful treatment of lung adenocarcinoma patients positive for SQSTM1-ALK with pericardial effusion using ensartinib. The outcomes indicate that ensartinib may be considered as an effective therapeutic option in the presence of SQSTM1-ALK. These findings hold significant implications for clinical practice, emphasizing the necessity of genetic testing and tailored treatment approaches for lung adenocarcinoma.

## Data Availability

The raw data supporting the conclusions of this article will be made available by the authors, without undue reservation.

## Abbreviations

ALK, Anaplastic lymphoma kinase; CEA, Carcinoembryonic Antigen; IMFT, Inflammatory Myofibroblastic Tumor; TTF1, Thyroid Transcription Factor-1; IMT, Inflammatory Myofibroblastic Tumor; SQSTM1, Sequestosome 1; TPS, Tumor Proportion Score.
